# Bow Your Head in Shame, or, Hold Your Head Up with Pride: Semantic Processing of Self-Esteem Concepts Orients Attention Vertically

**DOI:** 10.1371/journal.pone.0137704

**Published:** 2015-09-14

**Authors:** J. Eric T. Taylor, Timothy K. Lam, Alison L. Chasteen, Jay Pratt

**Affiliations:** University of Toronto, Toronto, Canada; VU University Amsterdam, NETHERLANDS

## Abstract

Embodied cognition holds that abstract concepts are grounded in perceptual-motor simulations. If a given embodied metaphor maps onto a spatial representation, then thinking of that concept should bias the allocation of attention. In this study, we used positive and negative self-esteem words to examine two properties of conceptual cueing. First, we tested the orientation-specificity hypothesis, which predicts that conceptual cues should selectively activate certain spatial axes (in this case, valenced self-esteem concepts should activate vertical space), instead of any spatial continuum. Second, we tested whether conceptual cueing requires semantic processing, or if it can be achieved with shallow visual processing of the cue words. Participants viewed centrally presented words consisting of high or low self-esteem traits (e.g., brave, timid) before detecting a target above or below the cue in the vertical condition, or on the left or right of the word in the horizontal condition. Participants were faster to detect targets when their location was compatible with the valence of the word cues, but only in the vertical condition. Moreover, this effect was observed when participants processed the semantics of the word, but not when processing its orthography. The results show that conceptual cueing by spatial metaphors is orientation-specific, and that an explicit consideration of the word cues’ semantics is required for conceptual cueing to occur.

## Introduction

We regularly use space as a metaphor to talk about abstract concepts like self-esteem. This can be seen in phrases like “she held her head high with dignity” or “he bowed his head in shame”. We rely on spatial metaphors to provide a common, universally-understood metric for understanding [1]; they ground abstract concepts in the physical world [2]. If certain abstract concepts are grounded in spatial metaphors, then thinking of these concepts may activate a corresponding associated spatial simulation [3]. Consequently, this activation could affect the allocation of visual attention in a scene, an effect known as conceptual cueing [4]. In this paper, we use positively- and negatively-valenced self-esteem concepts as cues to orient attention, and investigate the conditions under which conceptual cueing occurs.

Conceptual cueing robustly demonstrates how concepts can orient attention by activating their associated spatial representations. The most concrete example of this is the use of direction words to cue attention; reading word cues such as “left” or “right” speeds subsequent target detection at meaning-congruent locations [5]. Other basic concepts without explicit spatial information can also cue attention. For example, we think of numbers on a mental number line: Small numbers are on the left whereas bigger numbers are on the right (the SNARC effect) [6]. When reading numerals at fixation, smaller numbers facilitate target detection on the left and large numbers facilitate target detection on the right [7]. In other words, numbers cue attention to the left or right (but see [8]). Another example of a deeply abstract concept causing conceptual cueing is time, where the past is represented by the left and the future by the right [9], [10]. For example, Weger and Pratt [11] found that reading words describing points in time (e.g., yesterday, tomorrow) speeds target detection on the side of space consistent with this spatial metaphor, indicating that the concepts cued attention. These time words act on attention like arrow stimuli [12].

Even more abstract than numbers and time, religious concepts and the concept of power can also orient attention. Centrally presented words relating to the divine cue attention up and to the right, whereas words relating to the chthonic cue attention down and to the left [13], consistent with metaphors employed in the Judeo-Christian and Islamic traditions [14]. And words describing positions of power, such as “king” or “lord”, cue attention up, whereas words describing subordinate positions, such as “slave” or “prisoner”, cue attention down [15], consistent with demonstrations that representations of vertical space are activated when thinking about power words [16]. In a similar study, participants decided whether a word cue was a denigrating or elevating term before detecting targets above or below the word. Targets were detected faster when their position matched the metaphorical position implied by the honorific [17]. These studies lend credence to the idea that an abstract concept such as self-esteem, which we use in this study, can also orient attention. It should be noted that we are considering conceptual cueing of visuospatial attention as separate from other forms of spatial congruity effects that are modulated by semantic processing (e.g. [10]). For our purposes, we consider any task where a briefly presented cue word or sentence differentially affect perceptual processing of targets presented at different regions in space as affecting visuospatial attention. We can subsequently make predictions about how spatially organized concepts, like the vertically-arranged notion of self-esteem, can draw attention up or down. While the relationship between spatial representation of the cued concept and the directional specificity of visuospatial cueing has been well established, other elements of conceptual processing have been left unexamined.

One such unexamined element is the level of processing required to elicit conceptual cueing of attention. If the cueing effects depend on the activation of a spatial simulation associated with a concept’s meaning, it is reasonable to expect that semantic processing of the word cues should be necessary to elicit cueing. Many experiments investigating conceptual cueing require that participants process the word cue’s meaning on every trial [4], [12], [13], [15]. However, other experiments simply had participants view the word cues passively, where it is unclear whether they processed those stimuli at a semantic level or not [7], [11], [18], [19]. One recent investigation measured attentional capture in a rapid serial visual presentation task to centrally presented words after semantic or visual processing of the words and found a prolonged attentional blink (temporal capture) only after semantic processing of the word cue [20]. This finding supports the idea that attentional capture by word cues requires semantic processing, but those word cues were not abstract, and the results speak to temporal, rather than spatial attentional capture. Given this dearth in the literature, in the present study we investigated the role of level of processing in abstract conceptual cueing of covert shifts of attention by directly comparing orienting on trials when self-esteem word cues were processed semantically (on a deep level)_or orthographically (shallow).

In this study, we used positively and negatively valenced self-esteem concepts to investigate the necessary depth of processing required to elicit conceptual cueing of the covert orienting of attention. Importantly, we manipulated the level of processing of word cues to directly compare the effects of implicit and explicit semantic processing on conceptual cueing. Specifically, participants completed a go / no-go detection task where targets appeared above or below centrally presented uninformative word cues. They made or withheld a response depending on the word cue’s meaning (semantic condition) or the visual features of the letters it was printed in (orthography condition). Unlike other experiments where word cue processing was deemed implicit because there was no instruction to process the meaning (i.e. implicit by default), our orthographic processing condition was designed specifically to direct the reader’s attention to the word’s visual features, rather than its meaning. Given that attentional orienting to conceptual cues is contingent on the activation of a spatial representation from a linguistic metaphor, we predicted that semantic processing of the word cues would be necessary to elicit the cueing effect.

Positively and negatively valenced words have been shown to elicit strong spatial congruency effects along the vertical axis. For our purposes, the most relevant demonstration of this spatial congruency was conducted by Meier & Robinson [21], who showed that positively and negatively valenced affect words (e.g., “happy”, “sad”) are processed faster when they are positioned in a congruent part of the display (e.g., top or bottom of the display, respectively). Here, we extend the use of valenced personality words to conceptual cueing to see if high or low self-esteem word cues would shift attention. Given the demonstrable association between verticality and valence [21] and also power [15], we expected self-esteem words, which are closely related, to also orient attention on the vertical axis. We also employed a condition where targets appeared left or right of the word cues; given the lack of horizontal metaphors for self-esteem concepts, we did not expect to observe a horizontal orienting response. However, valence has strong horizontal congruity effects [21], so if self-esteem is merely a type of valence concept, it should also cue attention horizontally. If self-esteem utilizes a distinct underlying spatial metaphor, then it should only orient attention vertically. If, on the other hand, self-esteem is really a nuanced expression of the valence embodied metaphor, we should also see cueing effects when targets appear on the horizontal axis, in accordance with existing demonstrations of horizontal priming by valence [21]. Including the horizontal condition, therefore, might allow us to disentangle the spatial representation of self-esteem concepts from valence in general.

## Methods

### Ethics

All participants provided written informed consent. This study was approved by the University of Toronto’s Institutional Review Board for ethical conduct of research with human subjects.

### Participants

Thirty-eight undergraduate students (11 male) from the University of Toronto participated in exchange for course credit.

### Materials and Stimuli

All stimuli were displayed in white on a grey background on a computer monitor. Head position was stabilized with a chinrest 54 cm from the display to control for head movements and to maintain a constant viewing distance. Word cues were presented centrally, in Arial size 28 font (~2.2° maximum height). Thirty of the words were adjectives describing high or low self-esteem traits (e.g., confident, timid), and thirty were filler words describing types of furniture (e.g., chair, cupboard; see Table 1 for a complete list of word stimuli). The target was a circle (2.0° diameter).

**Table 1:** List of word cues used in the experiment.

High SE Trait	Low SE Trait	Filler Words	
Confident	Timid	Armchair	Mantle
Proud	Ashamed	Bed	Mattress
Cheerful	Gloomy	Bench	Mirror
Assertive	Passive	Blanket	Nightstand
Determined	Discouraged	Bookcase	Ottoman
Brave	Cowardly	Cabinet	Pantry
Energetic	Apathetic	Chair	Pillow
Decisive	Passive	Couch	Rug
Talkative	Quiet	Cupboard	Rack
Outgoing	Solitary	Desk	Shelf
Composed	Anxious	Dresser	Stereo
Charismatic	Boring	Footstool	Stool
Persistent	Doubtful	Hammock	Table
Resilient	Pessimistic	Highchair	Wardrobe
Commanding	Ruminative	Lamp	Waterbed

To ensure that our self-esteem words were truly representative of positively- and negatively-valenced self-esteem, we pre-tested them in a pilot study in which 14 separate participants rated the words. Specifically, we asked them to rate the degree to which each word described someone high or low in self-esteem on a seven-point Likert scale, with responses ranging from one (low self-esteem) to seven (high self-esteem). Consistent with our categorization of low and high self-esteem words, participants rated the high self-esteem words (*M* = 5.87, *SD* = 0.48) as significantly higher in self-esteem than the low self-esteem words (*M* = 2.39, *SD* = 0.46; *t*(27) = 19.80, *p* < .001, *d* = 7.89).

### Procedure

**Fig 1:**
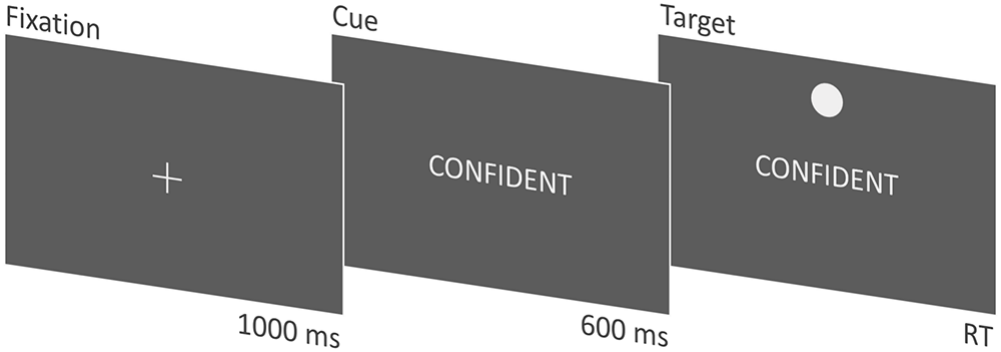
Time course for each trial. In this case, the target appears at the top location in the vertical condition.

Each trial began with a central fixation for 1000 ms (see Fig 1). Next, the fixation was replaced by a word cue for 600 ms, whereupon the target would be presented above or below (vertical condition) or left or right (horizontal condition) of the cue. The task was a go / no-go task with a single response. Participants were instructed to make a speeded response with their dominant hand. For “go” trials, participants were instructed to press the “H” button on the keyboard as fast as possible without making any errors. For “no-go” trials, participants were instructed to withhold a response entirely. The window for responses was 3000 ms. For the orthography condition, participants were instructed to respond to the target only if the word cue appeared in uppercase letters, and to withhold a response if the word cue was in lowercase letters. In the semantics condition, participants were instructed to respond to the target only if the word cue was an adjective, and to withhold a response if the word was a noun. Participants were informed at the start of each block if the following trials would involve either horizontally or vertically presented targets. The level of processing (orthography vs. semantics) and the orientation of the targets (horizontal vs. vertical) were within-subjects manipulations, blocked and presented in randomized order, for a total of four blocks. Within each block, every word was presented twice—once for each target position—for a total of 120 trials per block and 480 trials in total. Participants were encouraged to take short breaks after each block.

Self-esteem words were always presented in uppercase, while furniture words were always presented in lowercase. In this way, we ensured that participants were making responses to the self-esteem words while withholding responses to the filler words, regardless of whether participants were completing the orthography or semantic processing conditions. Feedback was given at the end of each trial, indicating whether participants appropriately responded or withheld a response. The experiment was programmed and run in Experimental Builder on a Windows PC.

## Results

Incorrect responses to no-go trials were rare (1.01%), and incorrectly withheld responses to go trials were rarer still (0.93%), confirming that participants followed instructions and processed the appropriate feature of the word cue (orthography or semantics). All further analyses were conducted on the trials of interest, where the cues were self-esteem words. Some RTs were recorded impossibly fast (less than 1 ms; 5.6%), so these trials were discarded. All remaining RTs below 100 ms (14.2%) and above 1000 ms (4.4%) were excluded from analysis to eliminate errors of apprehension and lapses of attention. The upper cutoff of 1000 ms was selected because it is often used in simple detection tasks and was very nearly 2 *SD*s (1 *SD* = 336.74 ms) from the grand mean (*M* = 369.35 ms).

**Fig 2:**
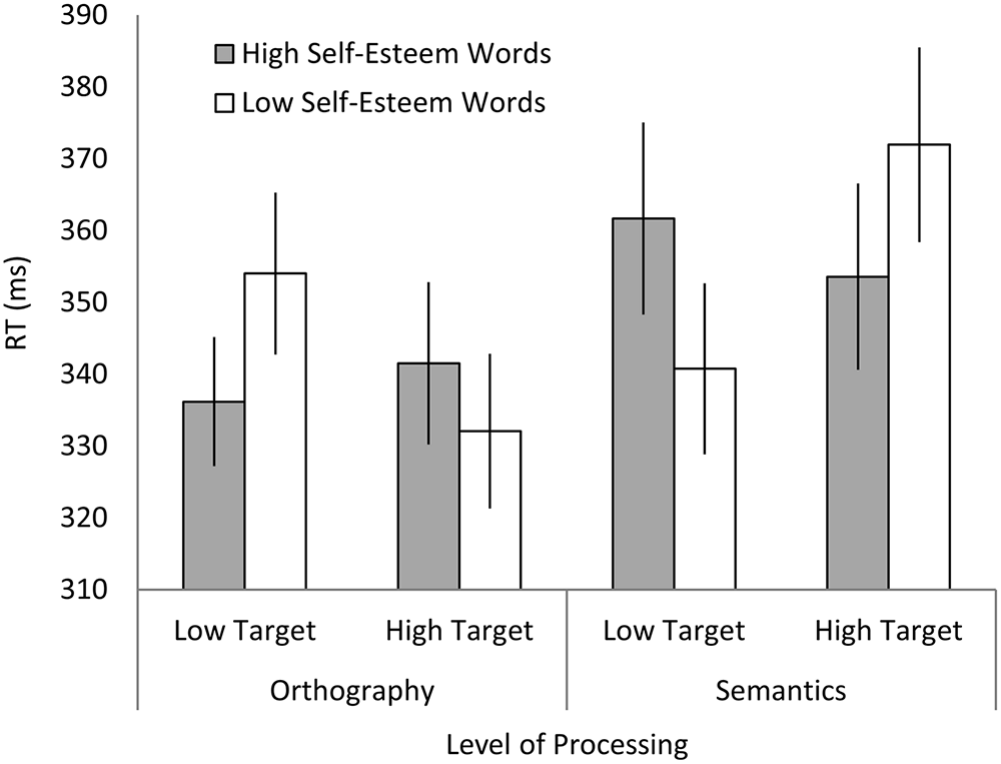
Mean RTs to detect targets in the vertical condition. Error bars represent within-subjects standard error.

For the vertical condition, where targets appeared above or below the cue word, mean RTs were entered into a 2 (self-esteem level: high vs. low) X 2 (target position: high vs. low) X 2 (level of processing: orthography vs. semantic) repeated-measures ANOVA. Critically, we observed the predicted three-way interaction: *F*(1,37) = 5.56, *p* = .024, *ɳ*_*p*_^*2*^ = .13 (see Fig 2). All other main effects and interactions failed to reach significance (all *F*s < 3.28, *p*s > .078). To probe the three-way interaction further, we conducted separate 2 (self-esteem level) X 2 (target position) ANOVAs for both levels of processing. When participants processed the semantics of the cue word, there was a significant interaction between target position and self-esteem, as predicted: *F*(1,37) = 5.23, *p* = .028, *ɳ*_*p*_^*2*^ = .12. Conversely, when they responded to the words’ orthography, there was no interaction (in fact it was trending in the opposite direction): *F*(1,37) = 1.92, *p* = .174, *ɳ*_*p*_^*2*^ = .05. Thus, the predicted interaction between self-esteem cues and target position only emerged when participants processed the meaning of the cue words, and not when they processed its visual qualities. The critical interaction’s effect size, *ɳ*_*p*_^*2*^ = .12, is large or medium-to-large by statistical convention [22], [23], and is comfortably similar to effect sizes reported by all studies reviewed in our introduction (except [19], whose effect sizes are exceptionally large).

For the horizontal condition, where targets appeared left or right of the cue word, we conducted the same three-way ANOVA as in the vertical condition. The only significant effect was an unexpected interaction between self-esteem and level of processing: *F*(1,36) = 7.31, *p* = .010, *ɳ*_*p*_^*2*^ = .17; all other *F*s < 1.91, all other *p*s > .176. Inspection of the means revealed that targets were detected slower when preceded by low self-esteem words in the semantic condition, but not the orthography condition (see Table 2).

**Table 2:** Mean RTs to detect horizontally presented targets. Values in parantheses represent within-subjects SEM.

	Level of Processing			
	Orthography		Semantics	
	Target Position			
Self-Esteem	Left	Right	Left	Right
High	360.54 (8.59)	352.71 (11.89)	335.38 (10.62)	356.15 (10.34)
Low	349.17 (11.53)	348.92 (9.15)	372.29 (13.07)	371.51 (11.93)

## Discussion

Peripheral targets were detected faster when their location was congruent with the level of self-esteem indicated by word cues, indicating that self-esteem concepts orient attention in a direction consistent with their common metaphors. Reading a high self-esteem word, such as “proud”, activated a spatial representation that biased the allocation of attention to the upper half of the display. This finding establishes self-esteem as an embodied metaphor capable of activating a robust spatial representation on the vertical dimension. Our results also highlight two important caveats for conceptual cueing effects.

First, the self-esteem cueing effect depended on the level of processing involved in reading the cue words. Prior investigations on conceptual cueing typically involve having participants make a decision based on the word cue’s meaning—what we referred to as our semantic condition (e.g., [11], [13]), but do not include a condition where participants process the word cue at a shallow level. Consequently, it remained unclear whether semantic processing was necessary to elicit conceptual cueing effects. One notable exception is the finding that semantic processing of day and month words orient attention left or right, but passive viewing does not [24], providing some evidence that semantic processing is necessary to elicit conceptual cueing. However, it is not clear what kind of processing passive viewing involves. To differentiate between semantic processing and active, shallow processing, we directly manipulated the level of processing of cue words with two conditions that demanded active processing at different levels. Judging a word’s orthography is an active process at a shallow level, so comparisons in performance between our orthographic and semantic conditions permit conclusions regarding levels of processing in conceptual cueing. The self-esteem cueing effect was observed only when participants considered the meaning of the word. When they made responses to the orthography of the words’ letters, the cueing effect was not observed. Stroop effects suggest that even an orthographic appraisal of the word cues should activate their meaning [25], [26]. Thus, implicit activation of self-esteem concepts is insufficient for the cueing effect. Instead, an explicit appraisal of the concepts’ meanings is required. Conceptual cueing effects seem distinct in this way compared to non-orienting conceptual congruency paradigms, which show that implicit reading of word meaning can elicit activation of congruent spatial representations [27], [28], [29].

Second, the self-esteem cueing effect did not manifest for horizontally-presented targets. This is important because, if self-esteem concepts are representationally distinct from valence—if they are vertically and not horizontally represented—we would predict a null effect in this condition, given the lack of horizontal self-esteem metaphors in common parlance. Thus, this condition provided a reasonable attempt to falsify our prediction. The null effect in the horizontal condition indicates a separation between the concepts of valence and self-esteem. Valence has strong associations (as demonstrated with spatial congruence paradigms) with the vertical axis [21], and also the horizontal axis [28]. If conceptual cueing follows the same principles of spatial activation as in these congruence paradigms, then positively and negatively valenced self-esteem words may have done the same. Contrary to this expectation, self-esteem words cued attention only on the vertical axis, suggesting that the spatial embodiment of self-esteem concepts is exclusively vertical, distinguishing it from valence in general. Furthermore, the horizontal condition also provides a control condition for the alternative explanation that conceptual cueing effects occur due to polarity correspondence [30]. In this view, participants can maximize performance in a speeded binary classification task (such as the one in the present study) by forming associations between response conditions (e.g., uppercase vs. lowercase) and response locations. This account can explain interactions in a speeded binary classification task [4]. It cannot, however, explain the null effect in the horizontal condition. It is theoretically possible that polarity correspondence might be differentially strong for the horizontal and vertical conditions, resulting in a response bias for the vertical but not horizontal conditions. However, our design has the further safeguard of using a single-button response, rather than a binary, two-location response, which makes binary, polar classification of responses in our task impossible. Thus, our results cannot be attributed to polarity correspondence.

The results from the present study enter into a growing literature regarding conceptual cueing effects on visuospatial attention. However, this growing literature also has some growing pains, as there are many inconsistent findings. When reviewing the literature on conceptual cueing of visuospatial attention, it doesn’t take long to find cases where conceptual cues variously incur costs or benefits to perceptual processing. For example, cues that facilitate target processing include: positive and negative affect (e.g., sincere or sour, [21]); power-related words (e.g., professor or student; [16], [15]); religious concepts (e.g., God or devil; [13]); sky or ocean words (e.g., eagle or dolphin; [31]); and, objects typically found in a specific location (e.g., sun or shoe; [32], [33]). Conversely, cues that have been shown to produce inhibitory cueing effects include upward or downward sentences (e.g., lizard ascended or cat descended; [34]); objects typically found in high or low locations (e.g., cloud or puddle; [19]); and motion words (e.g., leap or dunk; [35]). The fact that some conceptual cues cause facilitative effects when others cause inhibitory effects is not seriously problematic, as crossover effects are commonplace in visuospatial cueing: peripheral cues—the most basic visual cue we can imagine—differentially cause facilitation or inhibition depending on a number of factors such as stimulus onset asynchrony [36], or display elements [37].

Another candidate for the variable facilitation or inhibition caused by conceptual cues is the theory of event coding, which predicts that event features that share some dimension (e.g. the “upness” associated with positive self-esteem, and the location of the target) should prime responses at short delays, before they are bound into discrete events, and should inhibit responses at long delays, after the event is consolidated and the features are occupied [38]. Dissecting the reasons for these discrepancies is beyond the scope of this article. However, we will note that facilitative conceptual cueing effects tend to emerge when the task is shorter and involves relatively simple perceptual processing compared to tasks where inhibitory cueing effects emerge. Consistent with this observation, our results produced a facilitative cueing effect with a simple detection task and a brief stimulus presentation on par with original demonstrations of the symbolic control (i.e. using words as cues) of visuospatial attention [5].

In conclusion, this study establishes self-esteem as an embodied metaphor capable of affecting the spatial distribution of attention. Self-esteem can act as a conceptual cue for locations above and below fixation, suggesting these concepts are grounded in a vertical spatial representation. Furthermore, this study provides a direct comparison of the orienting of attention after deep versus shallow processing of word cue meaning, and shows that semantic processing of embodied word cues is sufficient to affect shifts of attention, but shallow orthographic processing is not.
